# Inclusive Parks across Ages: Multifunction and Urban Open Space Management for Children, Adolescents, and the Elderly

**DOI:** 10.3390/ijerph17249357

**Published:** 2020-12-14

**Authors:** Elin P. Sundevall, Märit Jansson

**Affiliations:** Department of Landscape Architecture, Planning and Management, Swedish University of Agricultural Sciences, P.O. Box 66, 230 53 Alnarp, Sweden; elin.sundewall@hotmail.com

**Keywords:** affordances, green space management, social multifunction, urban public space, user participation, user perspective

## Abstract

In urban areas where increased density has caused loss of urban open space (UOS), there is a need for high-quality parks that are inclusive and fit for multiple user groups. To make parks more inclusive, UOS management may need to consider multifunction and the perspectives of various age groups in future development and maintenance activities. Walking interviews were conducted in a park in central Landskrona, Sweden, with children, adolescents, and elderly users, and also with the head park manager of the city. The results revealed different perspectives among the three age groups of users concerning affordances and UOS management. The manager described user-oriented management to support multifunction and inclusion, including user participation. All user groups studied showed an appreciation of liveliness, contact with nature, social places for their own age group, clean and safe parks, and a variety of different atmospheres and activities in the park. Social multifunction can be developed in programmed or non-programmed ways, but some functions interfere with each other. UOS managers can develop parks to suit different age groups, promote user participation within management, and develop social multifunction to create inclusive parks for various age groups.

## 1. Introduction

Urban open space (UOS) provides multiple functions important for a city’s social, ecological, and economic sustainability. In cities with a growing population, densification is currently a common planning approach [1], but it can lead to fragmented, highly programmed UOS provision [2] or a reduction in the amount of UOS with increased user pressure, increasing the need for high-quality UOS [1].

The management of UOS is important for maintaining and developing its quality for the benefit of users [3,4]. However, despite general preferences of green spaces often focusing on accessibility, maintenance, nature, and facilities [5], perceptions of quality in UOS differ between user groups [6]. A park can be left unused by a certain group because it is perceived as unsafe or as belonging to another group [7]. In order to create a more inclusive public outdoor environment, all societal groups must perceive the environment as welcoming and have the opportunity to use UOS based on their needs and wishes. While greening of new areas is important for environmental justice, it might also cause new injustices due to increasing housing costs and gentrification of areas where some groups cannot afford to stay [7]. With focus on developing inclusive cities through management of existing spaces, different societal groups can instead get together in areas that meet several different needs.

Several research studies have investigated the ability of UOS management to adapt spaces for the needs and wishes of a specific user group, such as children [8] or the elderly [9]. However, few studies have compared different user groups, and similarities or differences between them, and little is known about how UOS management can contribute to inclusion of various user groups [10]. Multifunction has been proposed as a way of improving UOS quality in densified cities [1], but it is unclear how multiple functions can be introduced in the management of UOS in general, and concerning social values and different user groups in particular. Despite some focus on user participation in UOS management [11,12,13] and a general willingness among users to participate [14], there is limited knowledge on how to include a range of user groups in order to make participation socially sustainable.

User age is important for how parks are used [15,16] and for the perceived opportunities for use, so-called affordances [17]. The age groups recognised as major users of parks are children and the elderly, while use by adolescents has been found to be low [18], but is insufficiently studied.

Children appreciate a variety of different places and elements in UOS, including both informal (e.g., nature) and formal (e.g., playgrounds) [8,19]. Areas that are less planned and less maintained can encourage exploration and free play, and improve children’s contact with nature [8,20].

For elderly users, accessibility, proximity to green spaces and safety are important factors in UOS use [15,21,22]. Social contact with others [21,23,24], maintenance and cleanliness [9,22,25], contact with nature [22,23] and paths and seating [22,25] are also important to elderly users.

Use of UOS by adolescents is reported to be low [18]. According to Kaplan and Kaplan [26], young people have been described to take a “time out” from being in green areas, as interests such as developing independence and spending time with peers dominate. However, recent studies indicate opportunities to adapt UOS to better suit the interests of adolescents [18,27], often excluded or even designed away from public places [28]. Adolescents often appreciate opportunities to spend time alone or with friends [28,29,30]. Cleanliness and maintenance are also important to this user group [18,31].

Management for inclusion of different age groups requires more knowledge of the uses and perspectives of the groups, the benefits of multifunction and the scope for park managers to develop inclusive parks for several age groups. The aim of this study was thus to identify similarities and differences in UOS uses and perspectives of different age groups (children, adolescents, and elderly users) in urban parks and investigate how UOS management can achieve social multifunction and inclusion. Focus was on the following research questions:

What similarities and differences are there between the uses and perspectives of children, adolescents, and elderly people in an urban park? How can UOS management develop social multifunction and inclusive parks for these three age groups? How can UOS management achieve socially sustainable user participation by these three age groups?

### 1.1. Urban Open Space Management

Management of UOS is often described in terms of activities that both maintain and develop the spaces over time, focusing on their utility for people or users [3,32]. The social aspects are important, as UOS management should be user-oriented and take many user perspectives into account, such as age groups and interests [4].

Management can be divided into three strategic levels, namely policy, tactical, and operational [32]. The policy level is important for long-term development, as this is where visions and goals are evolved. The tactical level is where plans, budgets, and timeframes are set. Well-defined tasks are then carried out at the operational level.

Participation can occur at all strategic levels of management and is an important component of management [6,11,13], including for achieving sustainable development in UOS [32]. The benefits of user participation are, e.g., a better understanding of the needs of users among park managers, reduced maintenance costs [12], greater user interest in parks [33], and higher perceived quality of the outdoor environment [5,6].

### 1.2. Developing Multifunction

In order for parks to fit different uses and perspectives, and be inclusive for multiple age groups, they need to offer different functions, as developed through a user-oriented management approach [4]. ‘Multifunction’ has become a well-used concept in urban contexts, dealing with how to integrate and develop several functions in the same area, but many studies mainly consider combining ecological and social functions in green infrastructure [1,34,35].

In urban landscape contexts, multifunction has mainly been developed within planning and green infrastructure, as a way to use a particular site more effectively by intertwining or combining different functions in time or space [36,37]. Time-based multifunction combines several functions in the same place, but at different times [4,36], while spatial multifunction can be achieved through three different spatial arrangements: (i) tessellated multifunction separating various functions within a specific area; (ii) partial multifunction combining different functions within the same area, where one or more functions are dominant; (iii): total multifunction with a balance between different functions in the same area [37].

Shams and Barker [35] examined social functions and found that a greater understanding of different users’ preferences was important in order to develop multifunction. Yttri [38] concluded that the design of a park should reflect the different needs and interests of people in the area and provide many possibilities for use, as places programmed to suit specific user groups or activities may appear exclusive to others. There are thus several advantages of developing multifunction in UOS, but there is also a risk that including many functions within a defined area may cause conflicts. It is thus important to consider and evaluate synergies and trade-offs between different functions when developing multifunction [39].

## 2. Materials and Methods

### 2.1. The Selected Case

**Figure 1:**
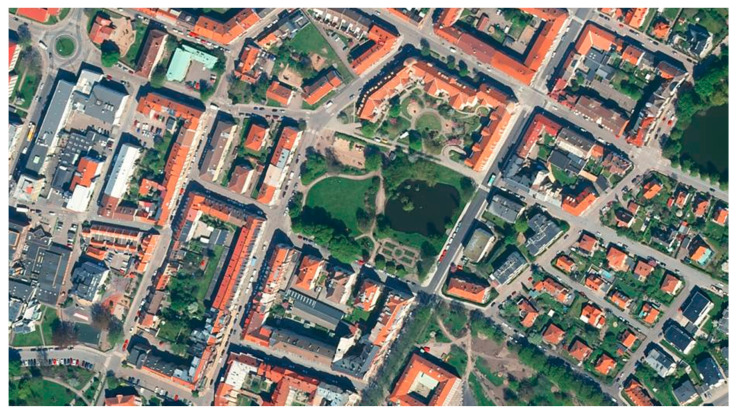
Wrangelska Parken and its surroundings. Background: GSD Orthophoto, 1 m colour © Lantmäteriet.

To investigate the perspectives of different user groups and of UOS management, a qualitative case study was conducted in autumn 2018 in Wrangelska Parken, a public park in Landskrona, southern Sweden. The city of Landskrona currently has around 45,500 inhabitants, but the number is increasing and densification is being used as a strategy for growth. The selected park is centrally located and comprises just over 1.5 hectares. The park is surrounded by small roads and a pedestrian and bicycle path. There are mainly apartment buildings around the park, but also a restaurant, grocery store, schools, and preschools within close range (see Figure 1).

**Figure 2:**
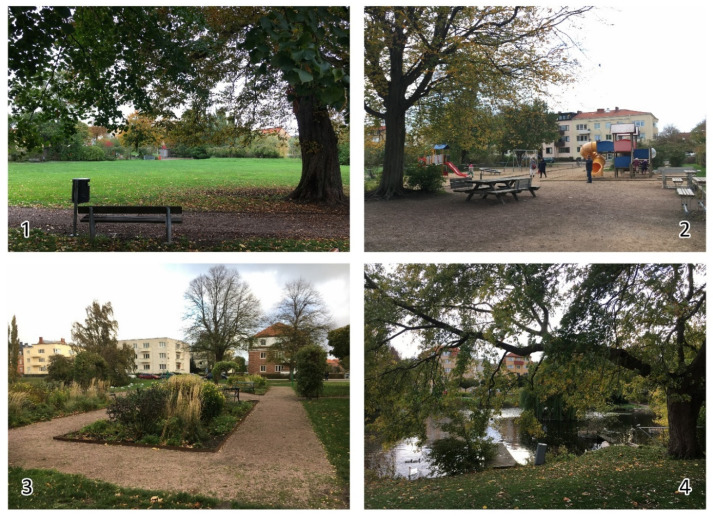
Parts of Wrangelska Parken. 1: open lawn area, with a path and a bench in the foreground, 2: playground with seating, 3: beds of perennials, 4: pond and bridge.

The park was selected for the study as it is relatively varied, with programmed places such as a playground, an area with perennial beds and benches, and walking paths, and also more non-programmed places, including an open lawn area with natural plantings, a pond with rich bird life, a fenced natural planting intended for bird life, and various types of vegetation such as shrubbery, ornamental trees, avenues of trees, and an evergreen plantation (see Figure 2).

### 2.2. Walking Interviews

The study was based on walking interviews with children, adolescents and the elderly, followed by a walking interview with the person responsible for the city’s UOS management. Walking interviews as a method can give interviewees greater control, having the opportunity to decide where to go and what they want to show the interviewer [40].

Semi-structured walking interviews were conducted with a total of 18 park users, in nine interviews (three for each age group, two respondents in each) (see Table 1). Children aged 9–10 and adolescents aged 15–16 were contacted in their schools, where information about the project was presented to them. Elderly users were approached directly in the selected park. Participating elderly people were aged 66–76 years. The aim was to include a broad spectrum of subgroups within each age group, with both sexes and with people with and without an immigrant background. However, the only adolescents interested in participating were female, while the only elderly users interested in participating, and able to understand the information about the study, were those with a non-immigrant background.

Oral and written information was provided about the research study and oral consent was obtained from all participants before the interviews. Written consent was obtained from the caregivers of all participating children and passive consent from the caregivers of the adolescents. Each interview lasted about one hour (range 30–84 min).

The interview method was developed and tested during two pilot walking interviews in a smaller park next to the selected park in spring 2018, one with adolescents and one with elderly users. The final interview guide comprised five themes: (desired) use, park content, atmosphere (calm/lively, other people), including/welcoming, and management/participation. A so-called “funnel-shaped interview” [41] (p. 94) was used, where each interview began with open questions and the interviewees were able to narrate in their own words and based on their interest. Over time, more specific questions were asked.

In order to gain a management perspective on social sustainability, multifunction and the results obtained in user interviews, the park manager responsible for operational UOS management in Landskrona local government was interviewed, using a similar walking interview approach as with the users. Questions in this interview concerned how management addresses social aspects in UOS, adaptation to and participation by users, and views on multifunction in parks. The uses and perspectives of different age groups were also discussed, based on topics raised during the user interviews.

All interviews were documented by audio-recordings and photos of places that the participants described or used during the interviews, supplemented with notes taken directly after each interview about features that the audio-recording could not capture, such as actions.

### 2.3. Analysis and Analytical Framework

The interview recordings were transcribed verbatim and analysed qualitatively, using the program NVivo 11 for thematic analysis [42]. The interview with the park manager was analysed at semantic level, while the user interviews were analysed at latent level [42]. The three age groups were first analysed individually, which led to unique categories for each group. A restructuring was then made based on a holistic view of the three analyses, forming two main categories common to all age groups: affordances and management.

The theory of affordances was used as an analytical framework, following the concept by J.J. Gibson in 1979: “The affordances of the environment are what it offers the animal, what it provides or furnishes, either for good or ill.” [17] (p. 119). Affordances are in one way objective and physical, whereas values are more subjective and mental, but are still not physical in that they can be measured objectively, because they always relate to the individual: “It is equally a fact of the environment and a fact of behaviour. It is both physical and psychical, yet neither. An affordance points both ways, to the environment and to the observer” [17] (p. 121). The design of a place is significant for what it can offer. Sites or things with a neutral design can have “multiple affordances” [43] (p. 254). For example, a traditional bench can offer something to sit on, but be perceived as unsuitable for other purposes, while a more neutral bench design can stimulate the creativity of the viewer and possibly offer more affordances, such as climbing on or using as a table. Gibson’s theory of affordances has been useful for investigating the functionality of environments for different user groups [29]. In the present analysis, the theory of affordances was applied by scrutinising the interview material for affordances the different age groups found in the park.

## 3. Results

### 3.1. Children and Affordances

**Figure 3:**
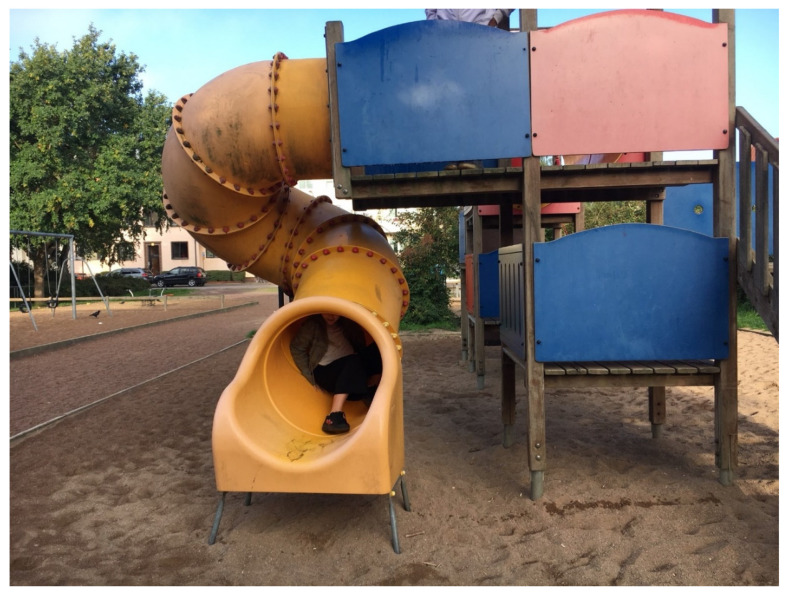
The slide and other equipment were popular among the children, as places to be active.

Children used the park much for *physical activity and play*. One boy said that the positive thing about being outdoors was to be able to run, use all the energy in one’s body, and breathe fresh air. Chasing and hiding games were popular activities in the park. Ball games and other sports were popular on the open area and some would have liked football goals there. The playground was useful for lively games (Figure 3), but the children expressed a desire for more challenging play equipment.

The playground was the first choice for all children to visit, described as a place where they could *socialise and play* with friends or get to know new friends. It was considered boring when no other children were there, but could also be too crowded, making children wait in line for their turn on the equipment. The children showed acceptance that the play equipment needed to suit younger ages, but wanted more exciting equipment for their age group and features in or next to the playground for their parents to appreciate. The children thought that the park as a whole should fit all people and be livelier, and they showed a general interest in other people present in the park.

The shrubbery between the playground and the open area was described by children as fun or exciting. The *play value of the vegetation* was high, with many affordances for fantasy play, a hidden spot for hide and seek-games and possibilities to manipulate and build dens. One girl described how she and her friend had once tidied up in the shrubbery: “here there were thorny plants that my friend and I cut away”. Some picked up loose natural material such as chestnuts, seed pods, and other parts of plants during the interviews. Some children described another playground, located in a forest, as more fun, where they could gather branches to build dens and find good climbing trees.

**Figure 4:**
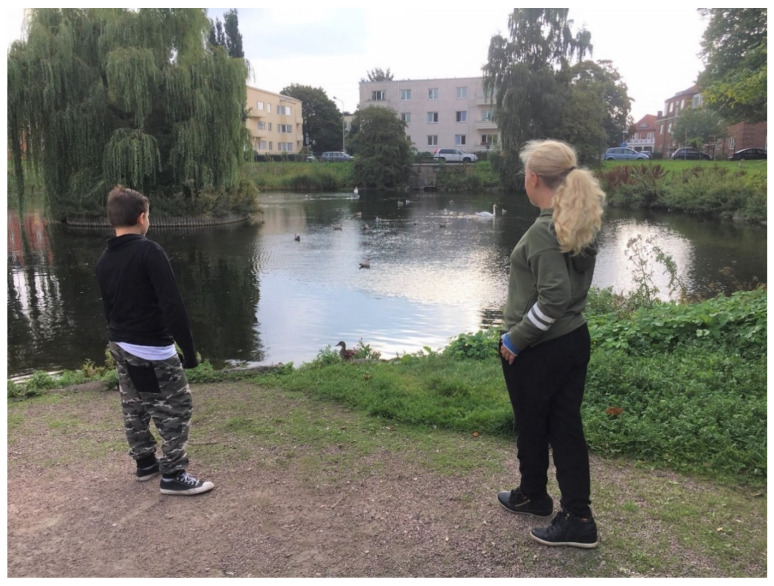
Several children were attracted to the pond to watch the birds.

*Nature contact* appeared important, as the children described several sensory experiences such as the sound of water, seeing leaves falling, feeling the warmth of the sun, and breathing fresh air. Nature also seemed to awake their curiosity. By the pond, described by a girl as one of the best places in this park, birds caught their attention (see Figure 4). During the interviews, some children stayed by the pond for a long time, watching the birds and talking about a swan family that lived there. Environmental issues such as climate change and water pollution were also brought up by many at the pond. Observing and experiencing animals and nature seemed connected to concerns about nature.

Places with *variation and combinations of different elements* seemed to be more attractive for children’s play. When the children described their favourite parks, they often talked about a combination of elements, such as the playground with plenty of vegetation all around or open spaces with vegetation areas, animals and built elements. One girl described her neighbourhood yard as her favourite place: “because there are a lot of places where you can ride a bike, (…) a lawn where very many play football and stuff, and then we have a playground, swings, so it is a lot of fun”. In Wrangelska Parken, the children said that they often played hide-and-seek games, preferably in the open lawn area, combined with vegetation and play equipment. The shrubbery in between the playground and the open lawn area was much used for play, possibly due to its placement between two other surfaces that attracted the children.

### 3.2. Children and UOS Management

Regarding *maintenance*, the children cared much for ‘their’ place, the playground. Broken parts or dirt on play equipment and litter on the ground, especially cigarette butts, made them claim that the playground was not cared for properly. They also showed great concern about the environment and animals in the park and viewed litter in nature as negative. Some children wanted “those who decide” to address the problem and remove the litter from parks, add more waste bins or find ways to make people stop littering.

Concerning *perceived safety*, most children felt safe in Wrangelska Parken, possibly because they knew the park well and often visited it with their school and after-school centre. However, few of the children seemed to visit a park without adults, except for the parks or yards very close to their homes. This could be due to safety issues such as unease about alcoholics or teenage gangs, especially when they occupied playgrounds. Some children suggested that there should be a guard at the playground to increase safety.

Several children expressed a desire to *participate* in playground development and came up with ideas on forms of participation. One suggested letting school classes have a vote when renovating playgrounds. Some were also positive about the idea of taking care of the park themselves, e.g., by cleaning, especially in the playground. Being invited to participate seemed an exciting idea to several of the children.

### 3.3. Adolescents and Affordances

One thing all adolescents pointed out was that there were too few places for them to *sit and socialise* with friends in parks. They seemed to have a clear picture of what they wanted, namely a place with several benches and tables that invited and enabled socialising. Many of them also mentioned the importance of surroundings being nice and cared for, preferably with some flower beds, trees or a nice view: “a perfect place then (in a) park for me would be by trees with benches”. In addition, the adolescents preferred locations away from playgrounds and residential buildings, so that they were not disturbed by ‘screaming children’ and were not disturbing others. They proposed benches and tables on the open lawn area, where the shrubbery in front of the playground served as a screen. Seating in quieter areas was also desired, to allow them to be alone or with just one friend.

Some adolescents reported that they sometimes went for walks in parks, but preferably in more natural areas where they could *relax and get away*. Vegetation and nice views were considered important. The vegetation around Wrangelska Parken was valued for shielding the noise of the city. The proximity to a relaxing place from school or home was seen as important for the opportunity to take a break and clear one’s mind. However, getting too close to nature was perceived as unpleasant by some and they described insects as “shabby”.

**Figure 5:**
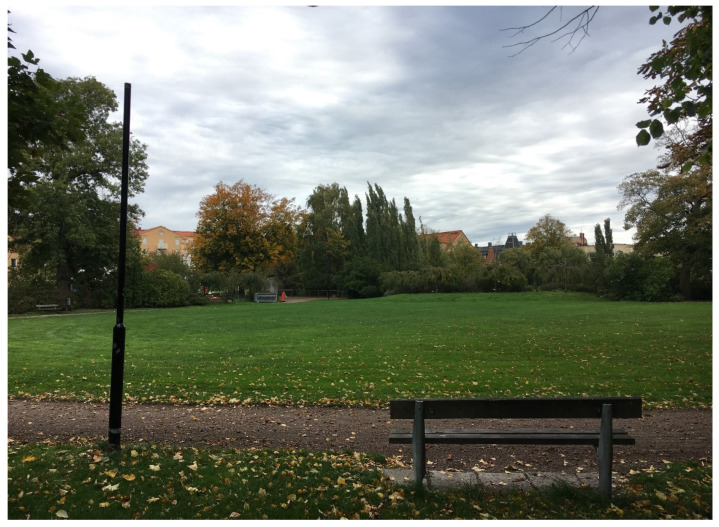
Adolescents wanted to have more activities in the open lawn area.

*Liveliness and activity* in parks was important for the adolescents and they reported that having others in the park made it feel both nicer and safer. The open lawn area was considered deserted, with nothing to attract people (see Figure 5). The adolescents wanted it to be used more by families and various ages and suggested a barbecue area where many could gather. Some also wanted to have more activity areas, such as an outdoor gym or a dance floor with music. Another park in Landskrona with a skate park and outdoor gym was described as lively and exciting. Cultural events such as outdoor cinemas, theatres, or creative places with the opportunity to, e.g., paint on a wall, were also considered attractive.

**Figure 6:**
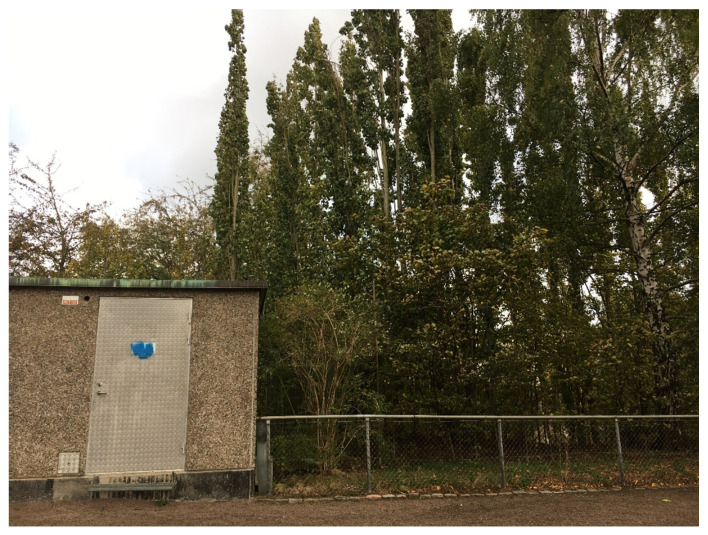
Adolescents suggested that the fence and electrical kiosk could be made more beautiful with vegetation cover or a happy colour.

*Nice looking* and well-kept places were considered important in parks and mentioned as a possibility to take pictures and post them on social media. By the natural planting, the adolescents showed a slightly greater acceptance of weeds, but instead mentioned the fence and the electrical kiosk next to it as boring and suggested that they could be masked by vegetation or painted in a happier colour to look more attractive (see Figure 6).

Developing “*cosiness*” with different types of seating seemed important, together with something nice to look at, such as perennials or water, and evening lighting. The adolescents also suggested dividing parks, using, e.g., shrubbery and trees, into different rooms to create different moods with more lively and quiet parts.

### 3.4. Adolescents and UOS Management

*Maintenance* was important for several adolescents. Weeds, litter, and dead animals were frequently mentioned as disgusting. The adolescents wished that the park could receive better care and argued that it would look better with many waste bins to reduce littering.

One reason for the desire to attract more people to the park was the *perceived safety* aspect. The adolescents sometimes felt unsafe and avoided parks that were hangout places for addicts or “boy gangs”. Some benches by the pond known to be often occupied by alcoholics were avoided, dense shrubbery where someone could hide was described as unpleasant and more lighting was proposed.

There was a desire among several adolescents to *participate* more in the community and some thought it was important for the local government to ask local people for opinions when developing parks: “You should kind of ask more people what they think should go where, instead of getting inspiration from another place that is modern and has a nice look”. There was a certain distrust in the local government and perceptions of UOS planners and managers wasting money when not consulting or listening to people before making changes. The interviewees thought that adolescents could be involved through the school, to share their views. A few were also positive to the idea of helping out with maintenance.

### 3.5. Elderly and Affordances

The elderly described it as a *pleasure to get out into the green* and appreciated having many parks preserved in the city. They paid attention to the vegetation and different species, especially the perennial beds and the flowering shrubs in between the playground and the open area, but also seemed interested in wild species in the natural planting. They often strolled in the park with a friend, partner, or dog. Some appreciated sitting and enjoying the sun or a beautiful view, seeing the bird life in the pond or taking a break under a shading tree.

*Liveliness* was described as important in the park, with enjoyment in watching children playing or others using the open space. The elderly appreciated visiting the playground with their grandchildren and one woman had used the open lawn area for picnics with her children and grandchildren. Others wanted the open area to be used more because it felt desolate, and suggested activities that could be arranged there, such as a multicultural food market—reflecting the multicultural area.

**Figure 7:**
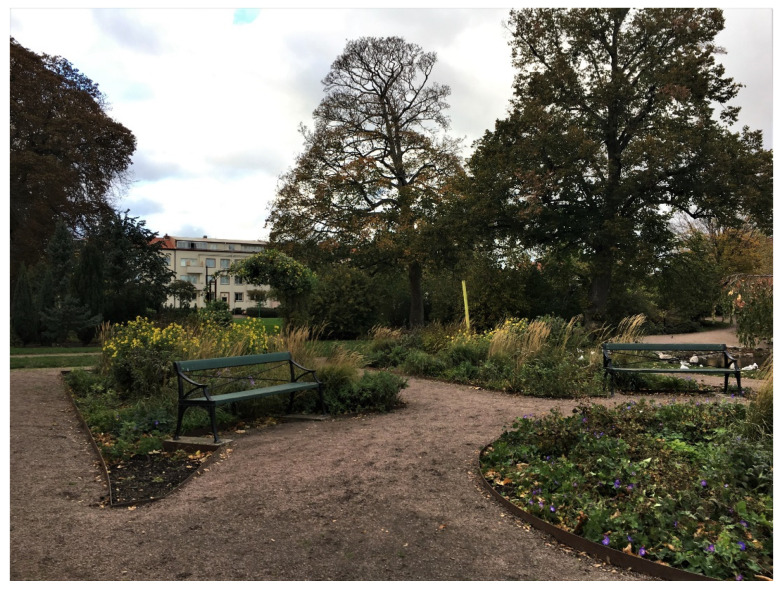
Seating by the perennial beds used for spontaneous meetings among the elderly.

The perennial beds with nearby benches were described as a *meeting place* for the elderly living around the park (see Figure 7). Many elderly people sat there and the interviewees reported that they often stayed there to talk to each other or enjoy the flowers.

Several described the importance of *aesthetic* parks with nice plants and views along the walkways. Some also wished parks to be more diversified to allow choices, with certain parks offering specific activities and seating for different occasions. Those who often visited the park and had good knowledge of it noted that it had decayed in recent years as, among other things, the perennial beds were not completely covered with plants.

It seemed that the elderly *connected to the park through memories* of their lives. A connection to their childhood was observed when they saw children in the park and recalled how they themselves had played in parks. Several compared Wrangelska Parken with how it was in the past and remembered more liveliness in the open lawn area: “When I was little there was a large stage there (…) so this was the meeting point, this was the best part of Landskrona”. The park was now perceived as more deserted due to removal of the open-air stage and other features. Positive changes were also mentioned, such as the removal of a fence around the pond.

### 3.6. Elderly and UOS Management

A well *maintained* park with beautiful and dense perennial beds, pruned shrubbery, and cleaned surfaces was desirable to the elderly users. They reported that the park was less cared for than previously and that other park users caused problems, such as littering and feeding birds, which had led to rat problems in the past year. However, in general, they were satisfied with the maintenance and showed acceptance for the less maintained natural planting.

For some older people, *safety* was the only aspect experienced as a problem in the park. Where shrubbery obscured the view and formed possible hiding places, more transparency was desired. All elderly respondents pointed out two places that they avoided, described as often being used by addicts or criminal gangs. However, presence of other people in the park was also described as a way to increase the perceived safety.

Few of the elderly were interested in *participating* physically in care of the park. This was attributed partly to bodily ailments, but also to disinterest. However, some elderly interviewees who lived next to the park showed extra care about it and had picked up litter and cut off branches that hung over the path. Several people were interested in sharing their opinions and ideas for how the park could be better utilised. Being consulted and having the opportunity to participate seemed desirable, but some lacked trust in the local government and said that their opinions would probably not be included even if they were given.

### 3.7. Park Management

The park manager was mainly concerned about technical solutions and ecological sustainability but reported that *social sustainability* was being taken into account more within the local UOS management. Areas with criminality problems were a current main focus, with emphasis on increasing safety in collaboration with the police, property owners, the social services department within the local government and a field group. Collaboration between these actors and responsiveness to citizens’ opinions were described as important in identifying and addressing problems. The park manager reported that parks with a poor reputation had been improved, with new perennial beds, walkways and trees, vegetation clearing, new lighting, and other equipment, and were now used more intensively. However, when removing shrubbery, a balance is needed with, e.g., biodiversity loss, and the park manager cautioned against making hasty decisions about clearing vegetation. The manager considered cleaning to be important in increasing the perceived safety and status in the neighbourhood, while also reducing people’s tendency to litter.

Opportunities for *citizens to share opinions* through different channels were considered important by the local government, according to the park manager. Opinions about safety have been collected through “safety walks”, aiming to identify places where perceived safety can be improved and to evaluate the measures. The city building department holds collaboration for officials to meet residents in areas affected by larger projects. There have also been invitations to residents to join management-related tasks, such as “clean-up days”, but a problem is that the same few people usually participate. The park manager expressed a need within park management to find out what residents want and who uses a park, before it is renovated, but had no answer on how more and other users can be involved.

The park manager believed that *multifunction* and the desires among the three age groups of users for both quietness and liveliness can be satisfied by dividing the park into different “rooms”, making the park more exciting. It was suggested that the open lawn area could be made livelier through collaboration with organisations that could arrange activities there. Parallels were drawn with another park in Landskrona, where several activities had been developed and which has now become very popular and appreciated by different age groups. Having parks that suit everyone, combining activities for different user groups at the same site, was considered important. In small parks, however, the manager believed that it is not possible to adapt to all different needs, so various functions should be made available in nearby parks. Having different features in different parks can stimulate curiosity among users and interest in visiting more parks in the city.

## 4. Discussion

### 4.1. Similarities and Differences between Age Groups

General perspectives regarding the content and use of parks were often similar for the three age groups, but there were also differences, particularly regarding how well the park and its affordances suited them. The elderly appreciated the park for walking and socialising, children thought that the park was quite fun, with the playground and a variety of places and affordances, and adolescents experienced the park as boring and empty, mainly because there was no place for them to socialise.

Despite the common desire among the respondents for parks to suit everyone, an appreciation for a place specially adapted for their own age group was expressed by interviewees. The advantage of such a place was mainly the opportunity to socialise with peers. While children used the playground and the elderly the perennial garden for socialisation, the adolescents lacked a place that suited them in this park, and in the city’s parks in general. Places for social interaction can be particularly important for young people, since social affordances motivate them to visit outdoor environments [29]. Places that are predefined for a specific age group may run the risk of being perceived as excluding other groups [38], which some adolescents seemed to believe when discussing what they are offered compared with younger children. Adolescents wanted their own place, preferably somewhat separate from playgrounds. This separation could also be appreciated by children, as they mentioned that adolescents near playgrounds can be perceived as unsafe.

Adaptation to users in UOS management was predominantly about safety measures. The safety aspect was described as important for parks to be inviting to users, especially the elderly. Informants in all age groups found that the presence of other people in parks provided a safe experience. This is thus important to take into account among the complex range of aspects affecting perceived safety in UOS, where the physical environment such as shrubs and lighting is important, but also individual and social aspects [44].

An appreciation of lively parks was common to all age groups, with the presence of other people contributing to attractiveness and to perceived safety and inclusiveness, as also previously found [35]. Many of the respondents described Wrangelska Parken as little used, which may be the reason for them desiring more liveliness. Other studies have noted that too few and too many park visitors are both undesirable [9,45,46]. The children described the disadvantages when the playground was crowded. Park managers should thus strive to develop liveliness in parks where use is low but avoid crowding.

An appreciation of nature and restorative environments was reported, especially by elderly users and adolescents. The restorative effect of nature in many ages has been demonstrated in several studies [45,47,48]. A shift has been noticed in users’ preferences in UOS from landscaped and well-managed to more natural, possibly due to increased public awareness of the ecological values that more natural vegetation provides [35]. For children, serenity was not mentioned as a reason to visit parks. However, they showed great interest in playing in a more natural environment and an appreciation for nature through their sensory experiences and interest in animals and plants. Green environments’ restorative effect on children [48] may contribute to children’s appreciation of nature. Knowledge about the ecological and restorative benefits of natural environments is important for park managers and for users. An important management task is thus to develop more vegetation and increase awareness of its values.

All user groups studied also expressed a desire for a clean park without litter, which is in line with previous findings [9]. A clean park signals a welcome and care to the users. Ensuring good cleaning is therefore a high priority, as also emphasised by the manager. This could involve identifying places with a greater need for cleaning, and devoting more effort there, or increasing users’ knowledge about ecological values to reduce littering.

A combination of elements and functions was appreciated by all three age groups. A combination of, e.g., play equipment, vegetation, and animals seemed to offer children more play opportunities, which was also in line with the park manager’s opinion. Similar results have been reported in other studies on children’s and adolescents’ outdoor environments [8,30]. Adolescents and the elderly also wanted different characteristics and content in different parks, which the park manager believed could contribute to greater interest among users in visiting different parks.

### 4.2. Developing Multifunction and Inclusiveness

The programmed places and objects in the park, such as the playground, the bridge over the pond and the fence, had value for those who found affordances there. Multifunction can be developed in these places by adding other affordances. In the playground, which the children found mainly suited to younger children, more numerous and more varied affordances can be developed to attract a wider age span, including something suitable for parents. According to Refshauge et al. [49], an appealing playground environment for parents, with opportunities to socialise with other adults and a variety of different play equipment, can encourage visits and longer stays with their children. Variation in playgrounds is thus important for both children and adults. The adolescents noted programmed objects in the park that were not intended for users such as the bridge by the pond and the electrical kiosk and suggested how these could be made more attractive to users. In this case, multifunction can be developed by combining functions intended for practical purposes with functions that can be used and appreciated by park visitors.

Wrangelska Parken also contained non-programmed places, like the open lawn and the shrubbery between it and the playground. Such places can offer several values because these affordances can stimulate visitors’ creativity [43] and because they afford different possibilities for use [38]. For the open lawn, which many informants described as desolate and empty, there were several suggestions on how to attract more visitors or uses, like soccer goals proposed by the children, a food market by the elderly, and a dance floor by the adolescents. This indicates that the open area, as well as other non-programmed areas, can stimulate the creativity of the visitor and that there are opportunities to use such sites more. Open spaces can be better utilised by developing time-based multifunction [36], where users are given the opportunity to arrange activities or manipulate the site in temporary arrangements.

The shrubbery between the playground and the open area appeared to be an example of total multifunction [37] for various ages, as children used it for exploring, hiding and for fantasy games, the elderly appreciated the beauty of its flowering shrubs and adolescents liked the serene atmosphere there, with the shrubbery shielding the open area against the playground. The multifunction suited different groups in different ways, showing that vegetation can offer a large range of affordances and that shrubbery with well-designed placement, plant selection and maintenance can create total multifunction in a small area.

There are thus many advantages of combining different functions, but also a risk of these functions interfering with each other [39], as confirmed in this study. Adolescents’ and older people’s appreciation for aesthetically pleasing, well-maintained vegetation is not fully compatible with children’s appreciation for less-maintained vegetation that affords various forms of play, e.g., creative play and exploration of the environment [8,20]. Shams and Barker [35] therefore suggest that UOS should move towards becoming more natural environments, without being too disconnected from the urban context. There were also contradictions among user groups in the desire to preserve or renew places. Adolescents described an appreciation for design changes, while elderly users appreciated things that are preserved, with a connection to the site’s history or appreciated uses. When developing parks, park managers should thus carefully consider the needs of different groups before making changes. This also concerns the balance between peaceful and lively activities. Tessellated multifunction [37] by, e.g., dividing parks into differently lively rooms, can be a way to handle the preferences of different user groups.

### 4.3. Developing Socially Sustainable User Participation

The general interest in participating in planning, management, and design of UOS found in this study, mainly among children and adolescents, but also some of the elderly, is in line with previous findings [14]. Young people have been found to appreciate participation in park projects, with the positive experience also leading to increased interest in outdoor environments and community involvement [33]. Problems with few participants and imbalances in opinions expressed were reported by the park manager and have also been noted by Molin and Konijnendijk van den Bosch [12]. Thus, there is a need to identify groups that rarely participate and target invitations at them, in order for participation processes to be more inclusive, above all groups that have less knowledge of the opportunities available to express opinions, such as children and adolescents. Invitations can make individual and site-specific needs and wishes visible [22], as suggested by adolescents in this study. Offering a variety of possible ways to participate, so-called mosaic governance, can also enable participation by different user groups [13]. Based on the interest in participating found in this study, participation in management should also take place at both operational and tactical levels.

### 4.4. Method Discussion and Future Studies

Studying three different age groups in the same park made it possible to compare their uses and perspectives and find similarities and differences, with the walking interview format providing site- and context-bound information. Interviewing the park manager provided insights into the work and experiences and gave ideas on how to develop social multifunction in the park. Weaknesses of the study were the low number of informants and the uneven distribution in terms of gender and geographical background among the adolescents and elderly. While walking interviews in pairs were well-functioning in allowing room for in-depth descriptions and discussions, conducting more interviews to include more respondents could increase the reliability of the results. Additionally, including more employees at different strategic levels of UOS management could give a more complete picture of the possibilities for management to address social issues.

The qualitative, on-site approach used in this study gave focus on the views of individuals of various ages. The insights gained might serve as a basis for more large-scale research studies using also quantitative or mixed-methods approaches, possibly questionnaires, for an overview including more informants. Future research could also focus on age groups throughout the life span, including different subgroups, and possibly include several groups together in, e.g., focus groups. Through intervention studies, different types of multifunction can be evaluated, such as the need for “own” places for specific user groups and opportunities to combine functions.

## 5. Conclusions

In densified cities with increased population, UOS management on policy, tactical, and operational levels is crucial to ensure inclusive parks. Better adaptation for different age groups, multifunction, and increased participation through targeted invitations to specific groups and broad mosaic governance approaches can be important measures. Social multifunction for different age groups is possible to a certain degree, as children, adolescents, and the elderly all appreciate having other people around, which can contribute to liveliness, increased appreciation, safety, and inclusiveness. In order to cater for several age groups, UOS management could consider the maintenance level of vegetation surfaces, preservation or renewal of places and development of lively and quiet areas. However, parks also need to offer places that attract specific groups and ages, providing opportunities to spend time with peers. Adolescents’ need for places in UOS that can satisfy their desires is particularly neglected.

## Figures and Tables

**Table 1:** Information about the respondents in the study.

Age Group	Interview	Gender	Age	Geographic Context
Children	1	Boy	10	Lived all life in Landskrona. Immigrant parents.
	1	Boy	10	Lived all life in Landskrona.
	2	Girl	10	Born in the region and mainly lived in Landskrona. Immigrant parents.
	2	Girl	9	Lived all life in Landskrona. Immigrant parents.
	3	Boy	10	Lives in Landskrona since age 5. Immigrant.
	3	Girl	10	Lived all life in Landskrona.
Adolescents	4	Girl	16	Born in Landskrona but lives in a nearby village since age 5. Immigrant parents.
	4	Girl	16	Lived all life in Landskrona. Immigrant parents.
	5	Girl	16	Born in Landskrona but lives in a nearby village since age 3. Immigrant parents.
	5	Girl	16	Born in other part of Sweden but lives in Landskrona since age 6. Immigrant parents.
	6	Girl	15	Lives in Landskrona since age 5. Immigrant.
	6	Girl	16	Lived all life in Landskrona. Immigrant parents.
Elderly	7	Man	76	Born in the region. Lives in Landskrona since 25 years.
	7	Man	69	Lived in Landskrona all life.
	8	Woman	75	Born in other part of Sweden. Lives in Landskrona since several years.
	8	Woman	66	Born in Landskrona and has also moved back.
	9	Man	76	Lived in Landskrona all life.
	9	Woman	70	Lived in Landskrona all life.
